# Highlighting the Importance of His Bundle Potential During Tachycardia and Ventricular Overdrive Pacing

**DOI:** 10.1002/joa3.70217

**Published:** 2025-11-10

**Authors:** Yasuharu Matsunaga‐Lee, Yasuyuki Egami, Koji Yasumoto, Masamichi Yano, Masami Nishino

**Affiliations:** ^1^ Division of Cardiology Osaka Rosai Hospital Osaka Japan

**Keywords:** atrioventricular nodal reentrant tachycardia, his bundle potential, orthodromic reciprocating tachycardia

## Abstract

Both AVNRT and NF‐ORT can be sustained with HV block. His–tachycardia dissociation during ventricular overdrive pacing is a key finding for differentiating AVNRT from NF‐ORT.

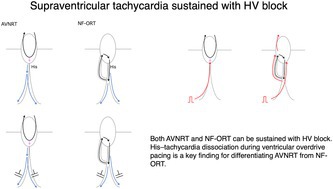

A man in his 10s was referred to our hospital because of symptomatic palpitations. The surface 12‐lead electrocardiogram (ECG) exhibited a narrow QRS tachycardia with a long RP interval. At baseline, the atrio‐His (AH) and His‐ventricular (HV) intervals were 73 msec and 56 msec, respectively. The effective refractory period of atrioventricular conduction during baseline pacing was 380 msec, determined by the occurrence of an AH block. A narrow QRS tachycardia was induced with ventricular burst pacing. During tachycardia, tachycardia sustained with atrioventricular block (AVB) (Figure 1 Left). The earliest atrial activation was at the proximal coronary sinus (CS). A V‐A‐V response was observed following ventricular overdrive pacing (VOP) from the apex of the right ventricle. The corrected post pacing interval minus tachycardia cycle length (cPPI–TCL) was 144 msec, as calculated 525–375–(97–91) (Figure 1 Right). The His‐atrial (HA) intervals during VOP and tachycardia were 258 msec and 249 msec, respectively. The difference between the stimulus‐atrial interval during VOP and the ventriculoatrial interval was 156 msec, calculated as 358–202. During VOP, the retrograde His potential was recoded just after the ventricular potential recoded at the His catheter (Figure 2). The stim‐His interval was stable after the first stable morphology of the QRS complex (SM), whereas the atrial‐atrial interval was reset after the third SM.

**FIGURE 1:**
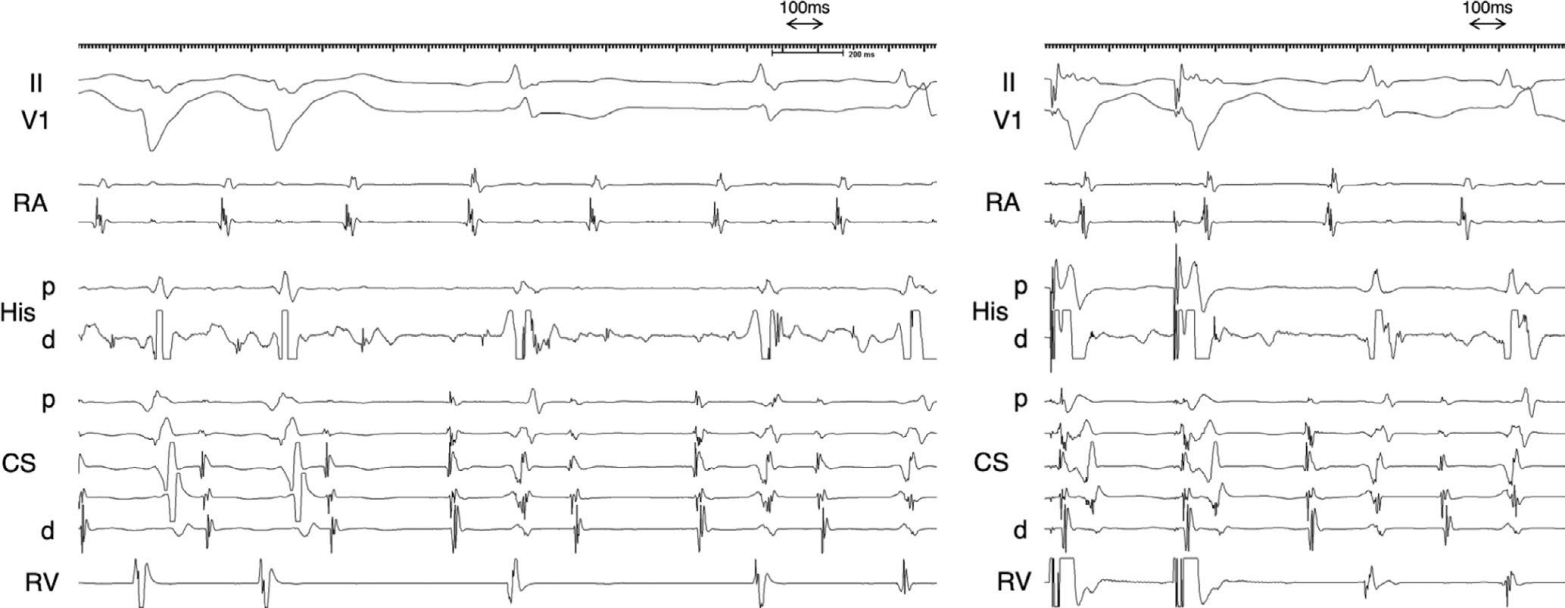
Left: Intracardiac electrogram during tachycardia with atrioventricular block. Right: Intracardiac electrogram at the cessation of right ventricular overdrive pacing. CS, coronary sinus; RA, right atrium; RV, right ventricle.

**FIGURE 2:**
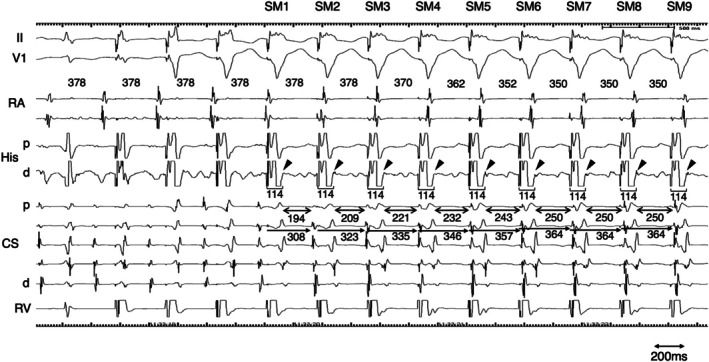
Intracardiac electrogram at the onset of right ventricular overdrive pacing. At the first stable morphology of QRS complex (SM), a retrograde His potential (black arrowhead) was recorded on the distal His catheter following the ventricular potential. Retrograde His potentials were also clearly observed from SM5 to SM9. The stim‐His interval remained constant after SM1. A double‐headed arrow indicates the His–atrial interval. SM, stable morphology of QRS complex. Other abbreviations are as in Figure 1.

Narrow QRS tachycardia persisting during AVB ruled out orthodromic reciprocating tachycardia (ORT) involving an atrioventricular or nodo‐ventricular (NV) bypass tract. A V‐A‐V pattern after ventricular overdrive pacing excluded atrial tachycardia. When AVB occurs at the atrio‐His conduction level, ORT using a nodo‐fascicular bypass tract (NF‐ORT) is also excluded (Figure 3). An atypical atrioventricular nodal reentrant tachycardia (AVNRT) could be sustained with AH block if a lower common pathway (LCP) is blocked. In this case, a His bundle potential was recorded during AVB, indicating that the block occurred at the HV level. Therefore, remaining possible diagnoses were atypical AVNRT or NF‐ORT (Figure 3). In this case, left and right bundle branch block morphology were observed during AVB (Figure 1). Although long PPI‐TCL supported atypical AVNRT [1, 2], the decremental property of the concealed NF bypass tract [3] could also result in a long PPI after VOP. In this case, a retrograde His potential was observed on the His catheter following the ventricular potential during ventricular extra‐stimulation at baseline. The conversion of the potential sequence from His‐ventricular to ventricular‐His was presumed to result from retrograde conduction block within the conduction system [4]. During VOP, a retrograde His potential was recorded on the distal His catheter following the ventricular potential after the first stable morphology of the QRS complex, and a stable stim‐His interval was subsequently observed during pacing. At the time when a His bundle potential was captured by VOP, the TCL, assessed by the atrial electrogram interval, was not affected by VOP. This observation was interpreted as His‐tachycardia dissociation, which is theoretically never observed in NF‐ORT. The tachycardia was diagnosed as an atypical AVNRT. Radiofrequency ablation at the earliest atrial activation site rendered the tachycardia noninducible, following a junctional response.

**FIGURE 3:**
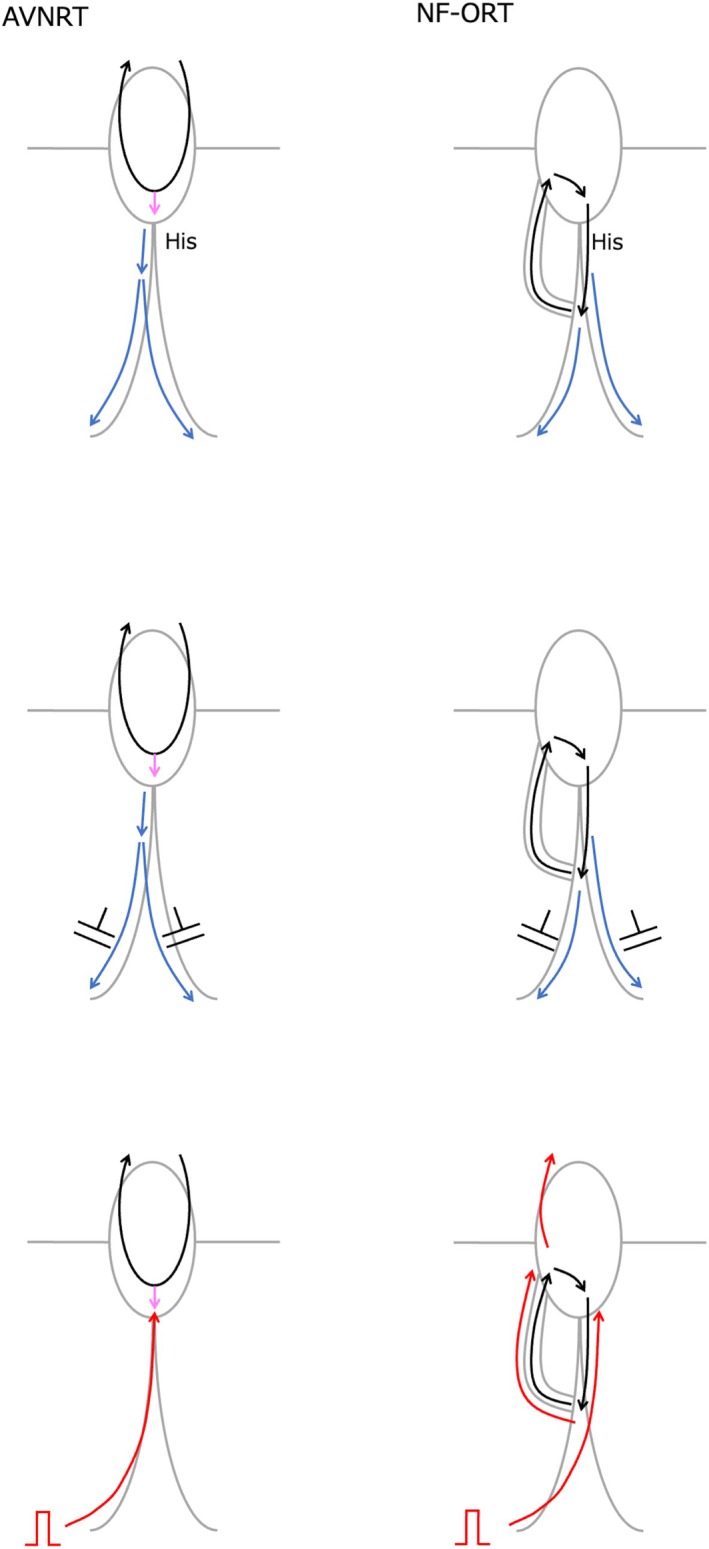
Upper panels: Schematic illustration of tachycardia maintenance during AV nodal reentrant tachycardia (AVNRT) and orthodromic reciprocating tachycardia using a nodo‐fascicular (NF) bypass tract (NF‐ORT). In this illustration, the NF pathway is hypothetically depicted between the right bundle branch (RBB) and the slow pathway. Middle left: AVNRT can be sustained despite AV block, either through AH block due to conduction block in the lower common pathway (LCP; pink arrow), or through HV block caused by bilateral bundle branch block (blue arrows). Middle right: NF‐ORT can be sustained despite HV block if a conduction block occurs distal to the NF bypass tract insertion in one bundle branch, along with a block in the contralateral bundle branch. Lower left: At the time of His reset during ventricular overdrive pacing (VOP), atrial reset is not observed if the pacing wavefront collides with the tachycardia wavefront at the level of the LCP. Lower right: At the time of His reset during VOP, the pacing wavefront penetrates the tachycardia circuit and results in atrial reset. The route of conduction to the atrium—via the His bundle or the NF pathway—may vary depending on the relative conduction times of the slow and NF pathways.

Narrow QRS tachycardia in the presence of AVB is often considered to represent either atrial tachycardia with AVB or AVNRT with LCP block. AVNRT with LCP block is associated with AH block. The presence of AVB at the AH level effectively excludes the possibility of NV/NF‐ORT. In the present case, however, AVB occurred at the HV level, necessitating further diagnostic evaluation to distinguish between AVNRT and NF‐ORT. Although NF‐ORT could theoretically be sustained despite an HV block if both left and right bundle branch blocks occur distal to the bifurcation of the NF accessory pathway, to the best of our knowledge, there have been no documented clinical observations of this in the literature. One possible reason is the diagnostic challenge in differentiating NF‐ORT from AVNRT. In a previous study that proposed diagnostic criteria for distinguishing NV/NF‐ORT from AVNRT, the cPPI–TCL was reported to be useful for NV‐ORT, but not for NF‐ORT [2]. This case underscores the clinical value of careful His bundle potential analysis in differentiating the underlying mechanism of sustained narrow QRS tachycardia in the presence of AVB. The theoretical concepts and limitations underlying the differential diagnosis during VOP are illustrated in Figure 4. The timing of the first reset His potential was defined as Hn. The preceding and following His potentials are defined as Hn‐2, Hn‐1, and Hn + 1. The interval between Hn‐2 and Hn‐1 corresponds to TCL, and the interval between Hn and Hn + 1 corresponds to the pacing cycle length (PCL). The interval between Hn‐1 and Hn is between TCL and PCL. In the case of NRT, since the His bundle is not included in the circuit, the timing of atrial reset can be delayed to the timing of His reset. The decremental property of retrograde nodal conduction, can prolong the HA interval. In this case, this decremental property accounted for the discrepancy of the atrial interval after His reset. In the case of NF‐ORT, since the His bundle is included in the circuit, at the timing of His reset, the tachycardia is reset by pacing, and the atrial interval should also be reset. However, because the interval between Hn‐1 and Hn is shorter than the TCL, the decremental property of retrograde conduction can mask the atrial reset. In this situation, the following His interval between Hn and Hn + 1 is even shorter than that between Hn‐1 and Hn, and the degree of atrial delay is expected to be even longer than the previous prolongation. Thus, the next atrial interval is speculated to be longer than the TCL. In this case, the atrial intervals were always ≤ TCL, which excluded the possibility of NF‐ORT. The limitation of this diagnostic interpretation arises when both the NF and slow pathways exhibit decremental properties. If these decremental properties act independently, a short interval input to the NF‐ACP can result in a longer input to the slow pathway, which in turn produces either a shorter or longer atrial interval or no net change at all. Taking these limitations into account, from the current electrophysiological standpoint [5], the most reasonable diagnosis in this case is AVNRT rather than NF‐ORT.

**FIGURE 4:**
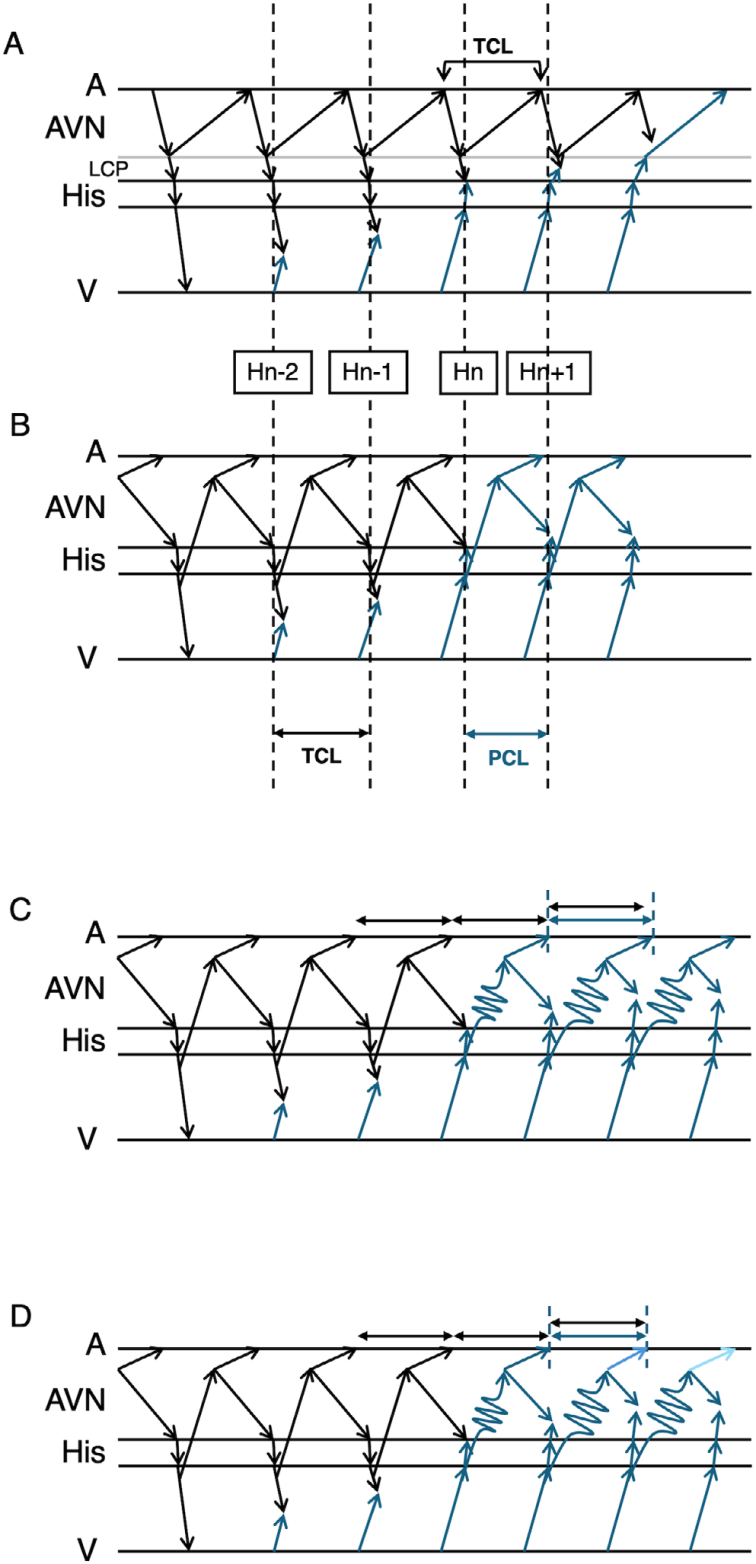
Schematic ladder diagrams illustrating the theoretical concepts of diagnostic interpretation during VOP. The first reset His potential is defined as Hn, and preceding and following His potentials are defined as Hn‐2, Hn‐1, and Hn + 1. The interval between Hn‐2 and Hn‐1 corresponds to the TCL, and the interval between Hn and Hn + 1 corresponds to the PCL. (A) Fast/slow atrioventricular nodal reentrant tachycardia (F/S AVNRT). The His bundle is not included in the circuit; therefore, atrial reset is delayed to the timing of His reset. (B) Nodo‐fascicular orthodromic reciprocating tachycardia (NF‐ORT). The His bundle is included in the circuit, and atrial reset is expected to occur with His reset. (C) NF‐ORT with decremental property of retrograde conduction. Because the interval between Hn–1 and Hn is shorter than the tachycardia cycle length (TCL), the decremental property of retrograde conduction can mask the atrial reset. In this condition, the following His interval (Hn–Hn + 1) is even shorter, and the atrial delay is expected to be further prolonged. Thus, the next atrial interval is speculated to be longer than the TCL. In the present case, atrial intervals were always ≤ TCL, which excluded the possibility of NF‐ORT. (D) NF‐ORT with individual decremental properties in both the NF and slow pathways. In this complex situation, the independent decremental behavior of each pathway could result in variable atrial intervals, either shorter or longer than the TCL, or unchanged. Taking these limitations into account, from the current electrophysiological standpoint, the most reasonable diagnosis in this case is AVNRT rather than NF‐ORT. NF‐ORT, orthodromic reciprocating tachycardia using a nodo‐fascicular bypass tract; NRT, nodal reentrant tachycardia; PCL, pacing cycle length; TCL, tachycardia cycle length; VOP, ventricular overdrive pacing.

## Conflicts of Interest

The authors declare no conflicts of interest.
